# Structural cartilage damage attracts circulating rheumatoid arthritis synovial fibroblasts into affected joints

**DOI:** 10.1186/s13075-017-1245-9

**Published:** 2017-02-28

**Authors:** Jan Hillen, Christiane Geyer, Marianne Heitzmann, Denise Beckmann, Annika Krause, Ina Winkler, Hermann Pavenstädt, Christoph Bremer, Thomas Pap, Adelheid Korb-Pap

**Affiliations:** 1 Institute of Experimental Musculoskeletal Medicine, University Hospital Muenster, Albert-Schweitzer-Campus 1, D3, 48149 Muenster, Germany; 2 Department of Anesthesiology, Intensive Care and Pain Medicine, University Hospital Muenster, Muenster, Germany; 3 Interdisciplinary Centre for Clinical Research, University Hospital Muenster, Muenster, Germany; 4 Department of Radiology, St. Franziskus Hospital GmbH Muenster, Muenster, Germany; 5 Department of General Internal Medicine, Nephrology, Hypertension Diseases and Rheumatology, University Hospital Muenster, Muenster, Germany

**Keywords:** Rheumatoid arthritis, Synovial fibroblasts, Transmigration, In vivo imaging, Mouse model

## Abstract

**Background:**

Rheumatoid arthritis synovial fibroblasts (RASFs) are known to travel via the bloodstream from sites of cartilage destruction to new locations where they reinitiate the destructive processes at distant articular cartilage surfaces. In this study, we examined the role of interleukin (IL)-1-induced cartilage changes and their chemotactic effect on RASF transmigratory capacity.

**Methods:**

To investigate synovial fibroblast (SF) transmigration through endothelial layers, we used a modified Boyden chamber with an endothelioma cell layer (bEnd.5) as a barrier and IL-1-treated murine cartilage explants as a chemotactic stimulus for SFs from human tumor necrosis factor–transgenic (hTNFtg) mice. We injected recombinant IL-1 or collagenase into knee joints of wild-type mice, followed by tail vein injection of fluorescence-labeled hTNFtg SFs. The distribution and intensity of transmigrating hTNFtg SFs were measured by fluorescence reflectance imaging with X-ray coregistration. Toluidine blue staining was performed to evaluate the amount of cartilage destruction.

**Results:**

Histomorphometric analyses and in vivo imaging revealed a high degree of cartilage proteoglycan loss after intra-articular IL-1 and collagenase injection, accompanied by an enhanced in vivo extravasation of hTNFtg SFs into the respective knee joints, suggesting that structural cartilage damage contributes significantly to the attraction of hTNFtg SFs into these joints. In vitro results showed that degraded cartilage was directly responsible for the enhanced transmigratory capacity because stimulation with IL-1-treated cartilage, but not with IL-1 or cartilage alone, was required to increase hTNFtg SF migration.

**Conclusions:**

The present data indicate that structural cartilage damage facilitates the migration of arthritic SF into affected joints. The prevention of early inflammatory cartilage damage may therefore help prevent the progression of rheumatoid arthritis and its spread to previously unaffected joints.

## Background

Rheumatoid arthritis (RA) is a chronic inflammatory disorder that leads to symmetrical polyarthritis characterized by synovial inflammation, cartilage destruction, and bone erosions [1, 2]. Research in recent years has identified synovial fibroblasts (SFs) in the lining layer of the hyperplastic synovial membrane as key player in the destructive process in RA [3]. SFs actively contribute to joint destruction by their secretion of matrix-degrading enzymes and inflammatory cytokines [3–6]. This aggressive phenotype is unique for SFs obtained from patients with RA (RASFs) [7]. Interestingly, RASFs retain their destructive potential in the absence of an inflammatory environment, as demonstrated by studies using mice with severe combined immunodeficiency (SCID) [8, 9]. In a recently published study using this SCID mouse model, it was shown that RASFs are more than just resident, immobile cells within the destructive synovial pannus. They have been suggested to contribute to the spreading of disease by their ability to leave cartilage destruction sites, migrate via the bloodstream, and reinitiate the destructive process at distant articular cartilage surfaces [10].

Because the ability of RASFs to enter and leave the bloodstream was demonstrated in a SCID mouse coimplantation model, it must be at least in part independent on a stimulated immune system. This suggests that not only cytokines implicated in immune processes but also other signals attract RASFs to cartilage surfaces and facilitate their transmigration at sites of extravasation. In this context, structural changes occurring in the articular cartilage early in the course of RA might be of importance as such signal for the diapedesis of RASFs. At present, very little is known about the early pathological changes within articular cartilage during RA, although it is widely agreed that biocellular changes precede the onset of arthritis development [11, 12]. Also, several lines of evidence suggest that cartilage damage, and particularly the loss of proteoglycans, precedes the attachment and activation of SFs at least in animal models of RA [13, 14].

On the basis of these data, we hypothesized that, in addition to alterations in the immune response, early cartilage damage is one important factor contributing to joints being affected by RA and potentially in the spread of the disease by attracting activated SFs into previously unaffected joints. The aim of this study was to substantiate this hypothesis by investigating the role of cartilage changes in mediating the transmigratory capacity of RASFs in a modified Boyden chamber assay and in an in vivo mouse model using fluorescent cell trackers. We obtained SFs from the human tumor necrosis factor–transgenic (hTNFtg) mouse model of RA and used interleukin (IL)-1 for the induction of intra-articular (IA) cartilage damage because it has been shown to initiate and mediate cartilage degradation in a mouse model of RA without affecting synovial inflammation [14–16].

## Methods

### Animals

C57BL/6 wild-type (WT) mice aged 12 weeks were used for the IA injection of cytokines and subsequent intravenous (IV) application of SFs. The injected SFs were obtained from mice heterozygous for the transgene of 3′-modified human soluble tumor necrosis factor-α (hTNFtg line Tg197, genetic background C57BL/6). These mice were generated and kindly provided by Dr. George Kollias and his group (Alexander Fleming Biomedical Sciences Research Center, Vari, Greece) [17]. For in vivo imaging and joint injections, inhalational anesthesia (Vetland Medical Sales and Services, Louisville, KY, USA) was performed by using 2.5% isoflurane with an oxygen flow rate of 1.0 L/minute. For ex vivo imaging and preparation of murine tissue, the animals were killed by cervical dislocation. The mice were bred in the animal facility at the University Hospital Muenster under standard conditions, and all animal procedures were approved by the local ethics committee.

### Tissues and cells

SFs were isolated from the hind paws of WT and hTNFtg mice. The skin and surrounding tissue of the hind paw were removed, and the synovial tissue was exposed to enzymatic digestion as previously described [18]. Cells were then cultured in DMEM (Sigma-Aldrich, Steinheim, Germany) at 37 °C in a 5% CO_2_ atmosphere. The medium was supplemented with 10% fetal calf serum (FCS; Biochrom, Berlin, Germany), 100 U/ml penicillin, and 10 μg/ml streptomycin (PAA Laboratories, Pasching, Austria). Cells at passages 3–5 were used for all experiments.

Isolation of murine femoral hip cartilage was performed as previously described [13]. In short, WT mice were killed at an age of 4–5 weeks, hip joints were dissected, and cartilage caps were isolated aseptically. Cartilage tissue was then cultivated in high-glucose DMEM containing 10% FCS, 100 U/ml penicillin, and 10 μg/ml streptomycin (PAA Laboratories, Pasching, Austria), 10 mM HEPES, and 50 μg/500 ml ascorbic acid for 24 h at 37 °C in a 5% CO_2_ atmosphere.

### In vitro transmigration assay

**Fig. 1:**
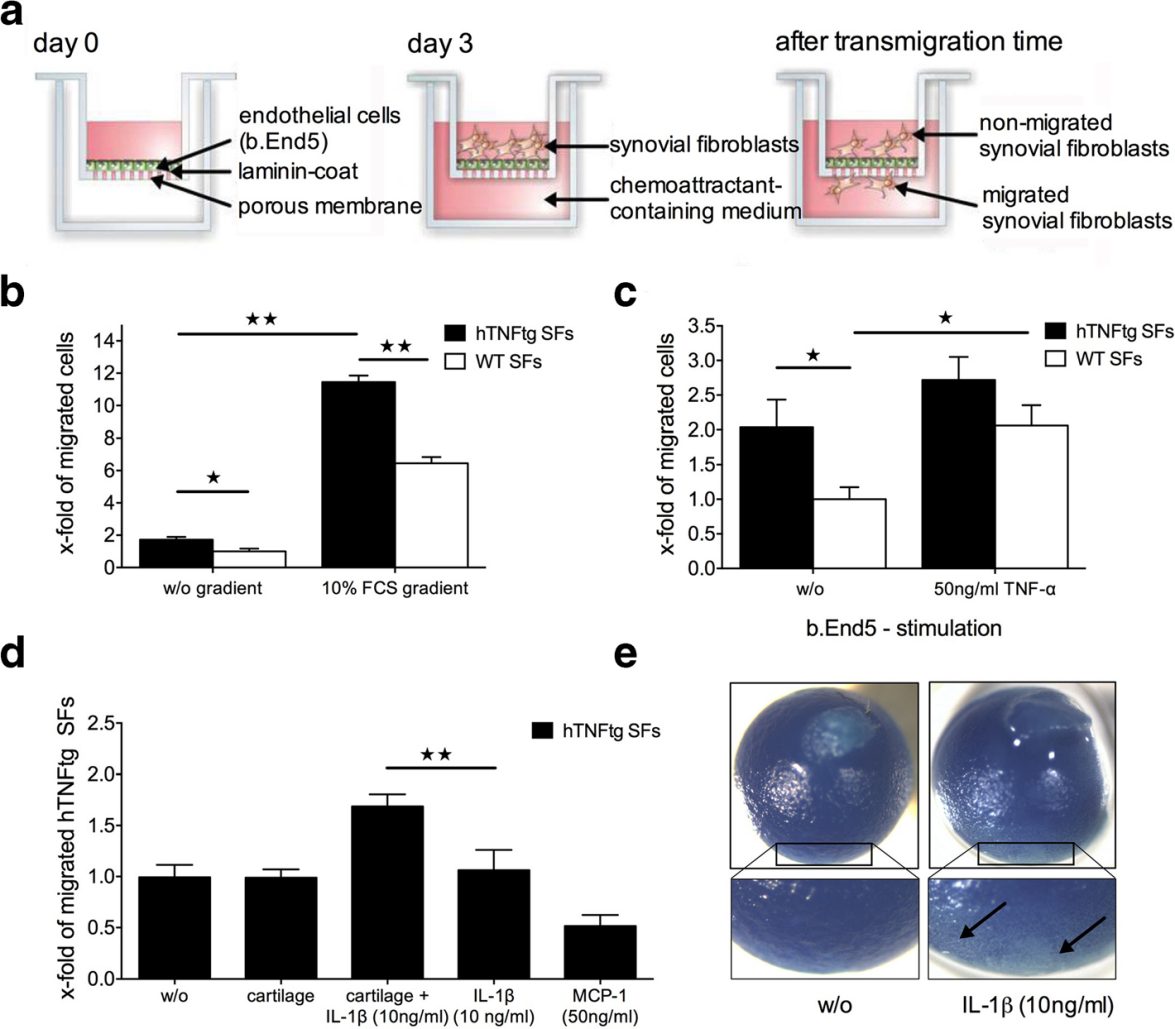
Cartilage coincubation with interleukin (IL)-1 stimulates transmigration of human tumor necrosis factor–transgenic (hTNFtg) synovial fibroblasts (SFs). **a** Schematic overview showing a modified Boyden chamber with a brain endothelioma 5 cell line (b.End5) layer on a laminin-coated porous membrane. SFs were seeded onto the b.End5 layer into the upper compartment, whereas the lower compartment contained different chemoattractant media. **b** Quantification of transmigrated SFs shows the significantly increased transmigration rate of hTNFtg SFs compared with SFs derived from WT mice (WTSFs), particularly when a fetal calf serum (FCS) gradient was generated. **c** Quantification of transmigrated SFs through an endothelial cell layer. hTNFtg SFs showed a significantly higher transmigratory capacity than WTSFs when migrating through an unstimulated b.End5 endothelium. TNF-α stimulation of the b.End5 layer prior to the seeding of SFs further increased the transmigration of both hTNFtg SFs and WTSFs, showing a significant increase for WTSFs. **d** Coincubation of murine cartilage explants with IL-1β in the lower compartment significantly increased the transmigration of hTNFtg SFs. In contrast, neither cartilage nor IL-1β alone was sufficient to enhance the transmigratory capacity of hTNFtg SFs. **e** Representative images of cartilage hip caps showing IL-1-induced proteoglycan loss (*arrows*) via toluidine blue staining. The values represent the mean ± SEM of at least three independent experiments consisting of duplicates as *x*-fold of (**b**) migrated WTSFs without gradient, (**c**) migrated WTSFs without b.End5 stimulation, or (**d**) migrated hTNFtg SFs without chemoattractant medium. *p < 0.05, **p < 0.01, Student’s *t* test. *MCP-1* Monocyte chemoattractant protein-1

To study the effects of cytokines and cartilage on the transmigration of SFs, an in vitro transmigration assay using a modified Boyden chamber was established (Fig. 1). For this purpose, cells from the blood-brain barrier (brain endothelioma 5 cell line [b.End5]), known for the formation of close tight junctions [19], were seeded and grown to confluence on a laminin-coated porous membrane (8-μm Transwell^©^; Sigma-Aldrich). Where indicated, the endothelium was stimulated by adding 50 ng/ml recombinant murine TNF-α (R&D Systems, Wiesbaden, Germany) into the upper compartment to facilitate the transmigration. TNF-α was removed after 4 h, and the SFs were seeded onto the endothelial layer in the upper compartment. Depending on the assay, the lower compartment was filled with medium alone (10% FCS gradient between the compartments) or with different stimulating agents such as 10 ng/ml recombinant murine IL-1β (R&D Systems), murine cartilage hip joint explants (three cartilage hip caps per well), or 50 ng/ml monocyte chemoattractant protein (MCP)-1 (R&D Systems), which were added to study their chemotactic effects on SFs. After a transmigration time of 16 h the SFs that had successfully migrated through the b.End5 layer were trypsinized from the basal layer and counted using the CASY^©^ cell counter (Roche Innovatis, Bielefeld, Germany). In vitro transmigration assays using different chemoattractant substrates were performed in duplicate in at least three independent experiments.

### Intra-articular cytokine injections

For the administration of cytokines, a volume of 5 μl containing 10 ng of recombinant murine IL-1β (R&D Systems) [15] in PBS or 5 μl of collagenase type IV (Worthington Biochemical Corp., Lakewood, NJ, USA) was administered by IA injection into the right knees as previously described [20]. Briefly, the region of the knee joint was shaved and disinfected. The joint was flexed at 90 degrees to provide access to the joint cavity at the lower pole of the patella. The injection was performed by puncturing the knee joint laterally from the patellar ligament using a 32-gauge microneedle (Hamilton AG, Bonaduz, Switzerland). The left knee joint was treated with the same volume of PBS to serve as a control. After the procedure, the animals were left for 48 h prior to IV injection of SFs to incubate the cartilage with the corresponding cytokine/PBS. For all IA injections, a stereomicroscope was used (Stemi 2000-C; Carl Zeiss Microscopy, Oberkochen, Germany).

### Fluorescent dyes for cell tracking

The lipophilic cell labeling solution 1,1′-dioctadecyl-3,3,3′,3′-tetramethylindotricarbocyanine iodide (DiR; Life Technologies, Carlsbad, CA, USA) was used to label SFs in vitro prior to their IV injection. The labeling was performed following the manufacturer’s instructions. To reduce dye transfer in vivo, cells were washed gently five times with PBS after being labeled, followed by overnight incubation and further washing before IV application.

### In vivo fluorescence imaging

**Fig. 2:**
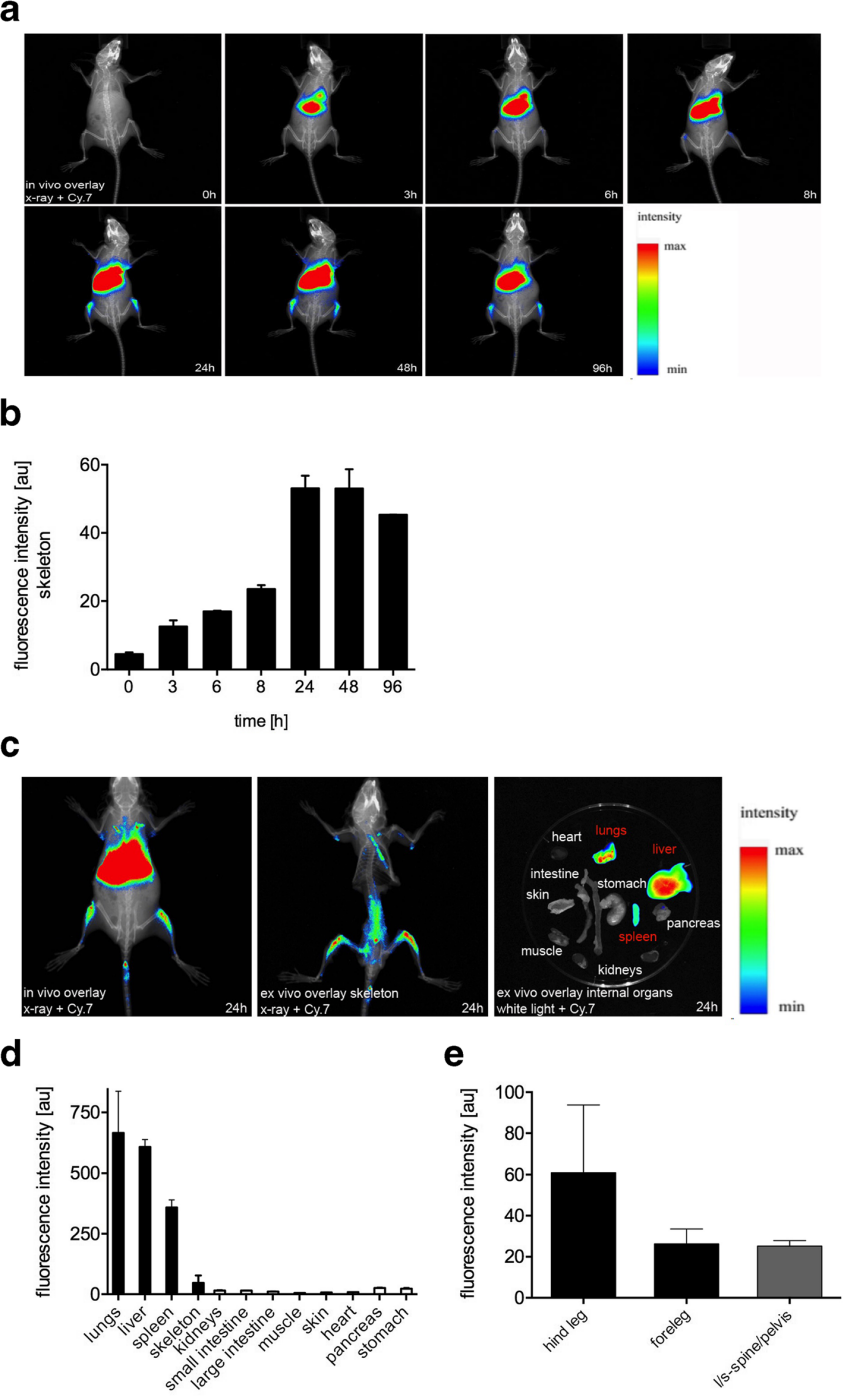
Tissue extravasation of intravenously injected 1,1′-dioctadecyl-3,3,3′,3′-tetramethylindotricarbocyanine iodide–labeled human tumor necrosis factor–transgenic (hTNFtg) synovial fibroblasts (SFs). **a** In vivo coregistration of X-rays and fluorescent signals (cyanine 7 [Cy.7] spectrum) showed an accumulation of hTNFtg SFs in the articular and periarticular zones of the hind legs and liver over time. **b** Quantification of fluorescent signals revealed an increase in intensity up to 24 h after intravenous (IV) injection of hTNFtg SFs in the regions of interest, such as the juxta-articular areas of femora and tibiae. **c**–**e** In vivo and ex vivo fusion images of the skeleton and internal organs with quantification of the fluorescent signals. At the time of maximal hTNFtg SF tissue extravasation 24 h after IV injection, detailed preparation and imaging of the different tissues revealed cells additionally in the lungs, liver, spleen, and the lower part of the spinal column and forelegs. The values represent the mean ± SEM for three animals. *l/s* lumbosacral

**Fig. 3:**
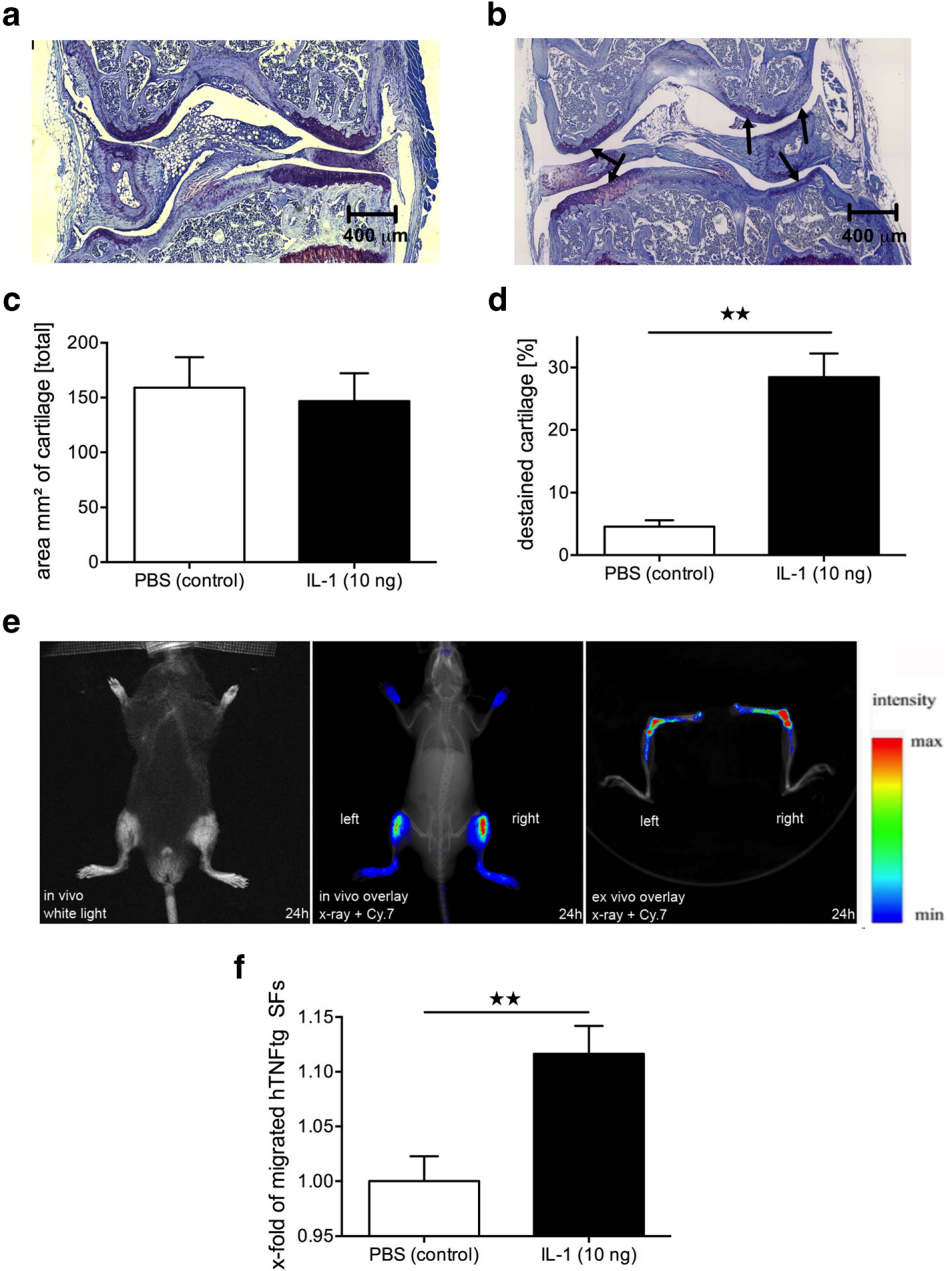
Interleukin (IL)-1-induced depletion of proteoglycans attracts human tumor necrosis factor–transgenic (hTNFtg) synovial fibroblasts (SFs) in vivo. **a**–**d** Wild type mice received simultaneous intra-articular injections of (**a**) PBS into the left and (**b**) IL-1 into the right knee joints. Forty-eight hours after injection, the cartilage of IL-1-treated knees revealed a significant loss of proteoglycans, as indicated by a higher percentage of destained cartilage (*arrows*) visualized by toluidine blue staining compared with control joints, whereas the total cartilage area was not affected (**c**, **d**). In contrast, the total amount of cartilage was equal in both groups. Scale bar = 400 μm. Mean ± SEM values are shown. ** *p* < 0.01, *n* = 4. **e** and **f** Intravenous injection of 1,1′-dioctadecyl-3,3,3′,3′-tetramethylindotricarbocyanine iodide–labeled hTNFtg SFs 48 h after intra-articular application of PBS/IL-1 showed significantly increased homing into the IL-1 pretreated knee joint. The values represent mean ± SEM for 12 animals. ** *p* < 0.01. *Cy.7* Cyanine 7

**Fig. 4:**
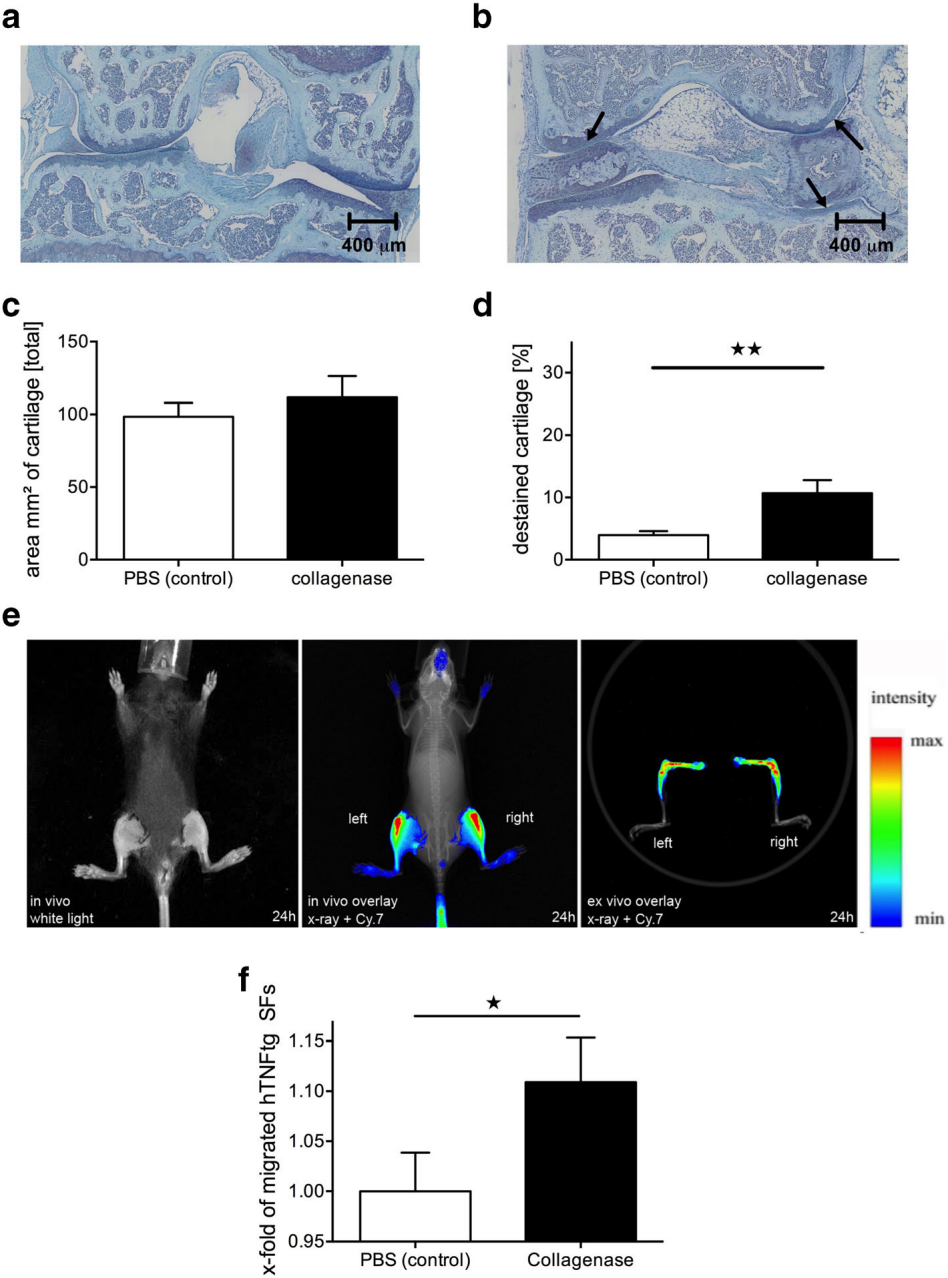
Intra-articular collagenase injection attracts human tumor necrosis factor–transgenic (hTNFtg) synovial fibroblasts (SFs) into knee joints. **a**–**d** Wild-type mice received simultaneous intra-articular injections of (**a**) PBS into the left and (**b**) bacterial collagenase into the right knee joints. **c** and **d** After 48 h, quantification of toluidine blue staining showed a significant proteoglycan loss (*arrows*) without affecting the total cartilage area. Scale bar = 400 μm, *n* = 4. **e** and **f** Subsequent intravenous injection of 1,1′-dioctadecyl-3,3,3′,3′-tetramethylindotricarbocyanine iodide–labeled hTNFtg SFs showed increased fluorescence intensity in the collagenase-pretreated right knee joint in comparison to the control left knee joint (*n* = 5). The values represent the mean ± SEM. * *p* < 0.05, ** *p* < 0.01. *Cy.7* Cyanine 7

In vivo fluorescence reflectance imaging (FRI) was carried out with the in vivo FX PRO Imaging Station (Bruker BioSpin MRI GmbH, Ettlingen, Germany). Mice were shaved using clippers and hair removal cream. A quantity of 1 × 10^6^ DiR-labeled hTNFtg SFs (100 μl of PBS with 50 U/ml heparin) was injected into the tail veins of WT mice using a 26-gauge needle. Images were taken at different time points, namely 0 h (before IV application) and 3 h, 6 h, 8 h, 24 h, 48 h, and 96 h after IV application. The fluorochrome distribution and signal intensity were measured over an image acquisition time of 30 seconds, followed by coregistration of x-ray and white light images for the creation of overlay/fusion images. The excitation light was set to 730 nm using an appropriate bandpass filter. According to an emission maximum of the DiR dye in the near-infrared spectrum at 790 nm, the fluorescent signal was detected using a 750-nm filter–equipped, high-sensitivity (4 million pixels) cooled charge-coupled device camera. After the last in vivo image was acquired, animals were killed for the removal of internal organs and the ablation of muscles and soft tissue from the skeleton, respectively. These samples were then imaged again separately to reduce interference and absorption by overlaying tissue sections. For the tissue distribution of hTNFtg SF (Fig. 2), WT mice were analyzed (*n* = 3), whilst for histological quantification and for the SF transmigration after IA cytokine injection (Figs. 3 and 4), at least three independent experiments were performed in each case. For the IL-1 and collagenase experiments, 16 and 9 mice were used, respectively.

### Histology and histomorphometric analysis

Knee joints of the WT mice were fixed in formalin (4%) overnight at 4 °C, decalcified in 10% ethylenediaminetetraacetic acid (Sigma-Aldrich) for 8 weeks and embedded in paraffin. For the evaluation of the articular cartilage and proteoglycan content, paraffin-embedded sections of the joints were stained with toluidine blue. The total amount of cartilage and the percentage of destained cartilage as a sign of proteoglycan loss were quantified as previously described [13].

### Statistical analysis

Data are expressed as mean ± SEM. Differences between the mean values of the groups were compared using an unpaired, two-tailed Student’s *t* test. *p* Values below 0.05 were considered statistically significant.

## Results

### SFs can cross endothelial barriers

To evaluate the general ability of SFs to migrate through endothelial junctions, an in vitro transmigration assay was established (Fig. 1a). First, we wanted to study potential differences in the transmigration rate between hTNFtg SFs and SFs derived from WT mice (WTSFs) independent of the presence of a barrier. Although only at very low levels, hTNFtg SFs showed a higher transmigration capacity than WTSFs (Fig. 1b) (+72% vs WTSF, *p* < 0.05). However, the generation of a 10% FCS gradient led to a massive increase of transmigrated SFs of both genotypes, particularly of hTNFtg SFs (Fig. 1b) (+77.5% vs WTSF, *p* < 0.01). Next, we performed these experiments using b.End5 cells as an endothelial cell layer. Although WTSFs as well as hTNFtg SFs were able to cross the endothelial barrier, the migratory potential of hTNFtg SFs was significantly higher (Fig. 1c) (+104% vs WTSFs, *p* < 0.05). Stimulation of the endothelial layer with TNF-α prior to the seeding of SFs further increased the transmigration of both cell types. Whereas hTNFtg SFs showed only a moderate increase (+34%, *p* = 0.22), the transmigration of WTSFs increased significantly after TNF-α stimulation (+106%, *p* < 0.05).

To investigate potential factors that further stimulate the transmigration of hTNFtg SFs through the endothelium in vitro, we investigated the role of articular cartilage as a chemoattractant for hTNFtg SFs. On the basis of the established function of IL-1 in inducing structural cartilage damage [14], IL-1β and murine cartilage explants were placed either alone or together into the lower compartment of the chamber. Neither IL-1 nor cartilage alone was sufficient to increase the transmigratory capacity of hTNFtg SFs (Fig. 1d). Interestingly, the coincubation of cartilage together with IL-1 led to a significant augmentation of hTNFtg SF transmigration through the endothelial layer into the lower compartment (+69.8% vs control, *p* < 0.01). As a control, cartilage hip caps were stained with toluidine blue, which revealed clearly destained areas following IL-1 incubation (Fig. 1e).

Following the evidence that preincubation of cartilage caps with IL-1 was an essential step in inducing SF transmigration, we also tested MCP-1, a known chemoattractant for SFs [21] as a transmigratory stimulus. For this purpose, MCP-1 was placed into the lower chamber (50 ng/ml); however, in contrast to the IL-1 experiments, MCP-1 did not stimulate SF transmigration (−49.5% vs control) (Fig. 1d).

### hTNFtg SFs migrate into joints within hours following their IV injection in vivo

To investigate the sites at which hTNFtg SFs leave the bloodstream and the time course of this process, we established an in vivo transmigration model. Therefore, hTNFtg SFs were stained with a lipophilic dye (DiR), followed by IV application and subsequent detection of the emitted fluorescent signal using an FRI chamber [22]. In vivo imaging revealed a strong signal associated with the large joints and adjacent bones of the hind legs (Fig. 2a). hTNFtg SFs homed into these areas within hours (3 h = 12.61 AU, 6 h = 17.00 AU, 8 h = 23.53 AU), showing the highest accumulation at only 24 h (53.06 AU) after IV application (Fig. 2b). After this time point, no further qualitative redistribution of the hTNFtg SFs to other tissues took place, and an apparent decay of the fluorescent signal was observed only several days after IV application (48 h = 53.03 AU, 96 h = 45.32 AU). For detailed analyses, the internal organs were removed 24 h after IV injection of hTNFtg SFs, and the skeleton was freed from muscles and connective tissue to reduce the absorption of the signal by overlying tissue sections (Fig. 2c). Ex vivo images showed hTNFtg SFs also in the lungs (665.22 AU), liver (608.50 AU), spleen (358.62 AU), spinal column (25.2 AU), and forelegs (26.3 AU) of the animals (Fig. 2d and e).

### IL-1 treatment leads to a loss of cartilage proteoglycans and stimulates the transmigration of hTNFtg SFs into the respective joint in vivo

On the basis of observations obtained from the in vitro transmigration assay showing that IL-1β-mediated cartilage changes stimulate the transmigration of hTNFtg SFs through an endothelial layer, we were interested in the role of predamaged joint cartilage as a chemoattractive stimulus for hTNFtg SF extravasation into the affected joint. Therefore, a single IA injection of 10 ng of IL-1β into knee joints of WT mice was performed. Histopathological analyses revealed a significant proteoglycan loss 48 h after IL-1 injection (Fig. 3a, b). The amount of destained cartilage indicating proteoglycan depletion was significantly higher in IL-1-treated knees compared with control knees (PBS) (28.5% vs 4.6%, *p* < 0.01) (Fig. 3d), whereas there was no significant difference in the total amount of cartilage (146.8 mm^2^ vs 159.0 mm^2^, *p* = 0.08) (Fig. 3c). No histological signs of inflammation were detected after IA injection of either IL-1 or PBS.

To determine whether this IL-1 treatment affected the attraction of hTNFtg SFs, we injected hTNFtg SFs intravenously following pretreatment of one knee joint with IL-1 and the other with PBS. Twenty-four hours after IV injection of hTNFtg SFs, FRI revealed a significantly increased homing of hTNFtg SFs into IL-1-treated knee joints compared with the PBS-treated control joints (+11.6%, *p* < 0.01) (Fig. 3e, f). Furthermore, ex vivo sagittal images of the joints showed a prominent signal in the knee joints, the distal femur, and the proximal tibia (Fig. 3e).

Because IL-1 not only induces cartilage damage but also constitutes an important inflammatory cytokine with the ability to affect chondrocytes or resident SFs in the joint in multiple ways, we also induced cartilage damage directly by collagenase application in vivo. In line with our IL-1 studies, we injected a single dose of 5 μl of collagenase solution into WT murine knee joints. Similar to the effects observed with IL-1, we found a significant collagenase-induced destaining of the articular cartilage, indicating proteoglycan loss (10.7% vs 3.5%, *p* < 0.01), whereas no differences in the total cartilage area were detected (111.72 mm^2^ vs 103.86 mm^2^). Twenty-four hours after DiR-labeled hTNFtg SF injection, an increased signal intensity indicating SF accumulation was detected in the collagenase-treated knee joints compared with the control knee joints (+10.9%, *p* < 0.05) (Fig. 4e, f).

## Discussion

Research conducted in recent years has revealed a number of mechanisms, such as abundant secretion of proinflammatory cytokines and proteases, that together perpetuate the inflammatory response in joints of patients with RA [23]. However, the ultimate cause of RA, as well as the changes during early stages of the disease that lead to the dissemination of arthritis to previously unaffected joints, remains largely unclear. In addition to systemic autoimmunity, SFs are of interest in this context because recent data derived from a SCID mouse model demonstrated SFs’ ability to migrate long distances and to reinitiate cartilage destruction at distant sites of subcutaneously implanted human cartilage [10]. Moreover, there is increasing evidence that in the course of disease, not only does cartilage purely suffer  damage but early changes to the cartilage in addition to immunological factors and mechanisms also contribute to fibroblast activation and thus to the switch from an acute to a chronic, destructive disease. In fact, several lines of evidence suggest that cartilage damage precedes the activation of SFs at least in animal models of RA, such as the hTNFtg mouse, and that prevention of IL-1-induced cartilage damage significantly alters the behavior of SFs in the course of this RA-like disease [13, 14].

In this study, we established an in vivo transmigration assay to visualize the tissue extravasation of hTNFtg SFs in a time-dependent manner. We found that circulating hTNFtg SFs migrate into joints and adjacent bones within hours following IV injection. Once the process of diapedesis from the bloodstream into the joint compartments was overcome, no significant quantitative or qualitative redistribution into other tissues took place. This emphasizes the primary function of SFs as resident cells in contrast to leukocytes that continuously recirculate between blood and lymphoid tissues as part of their function in immune surveillance [24]. Nevertheless, the attraction to and passaging of SFs at the endothelium-joint border requires close interactions between hTNFtg SFs and endothelial cells.

In vitro and in vivo studies of SFs and synovial endothelium from patients with RA have shown enhanced expression of adhesion molecules, including E-selectin, vascular cell adhesion molecule VCAM-1, integrin α_1_β_1_ (VLA-1), intercellular adhesion molecule ICAM-1, or junctional adhesion molecule JAM-C, resulting from the abundant secretion of TNF-α, because a therapeutic blockade with monoclonal antibodies effectively reduces the expression of these molecules [25–31]. In line with this, we found that the capability of SFs to break endothelial barriers in vitro is stimulated by the presence of TNF-α. Thus, both the endogenous expression in SFs (hTNFtg SFs vs WTSFs) and exogenous stimulation of endothelial cells with this cytokine led to an enhanced transmigratory capacity. This suggests an important role for TNF-α in the induction of adhesion molecules that are necessary for the transmigration of SFs through endothelial cell layers, although specific binding partners for this interaction are yet to be determined. However, hTNFtg SFs were also able to leave the bloodstream at sites of healthy, noninflamed joints of WT mice. This implies that chemoattractants other than TNF-α are also involved in this process. Interestingly, the joint compartments represent the only specific site of hTNFtg SF tissue extravasation because the transendothelial migration into liver, lungs, and spleen is most likely a result of their physiological function to purify and store cells from the blood.

Numerous data have shown that IL-1, another key cytokine in RA, mediates cartilage and bone degradation in RA [13, 16, 32]. In line with these data, we found that IL-1-induced proteoglycan depletion in cartilage hip caps as well as in knee joints of WT mice resulted in an increased transmigration of hTNFtg SFs in vitro and in vivo. In vitro only the coincubation of cartilage together with IL-1, but not IL-1 or cartilage alone, was able to stimulate the transmigration of hTNFtg SFs. The resulting notion that structural damage induced by IL-1 significantly contributes to the observed effects of IL-1 is also supported by our experiments in which the IA injection of collagenase resulted in effects similar to those seen with IL-1 treatment.

On the basis of these observations, we hypothesized that factors released from the joint cartilage as a result of cartilage damage are the important mediators that attract SFs into the joint and that structural cartilage damage increases the chemotactic effect on the transmigration of SFs. Although the exact nature of these factors remains to be established, there are a number of different possible mechanisms by which cartilage damage such as that induced by our experimental procedures as well as in patients with RA may lead to the attraction of fibroblasts into joints. These include the release of cartilage degradation products; the release of soluble factors that are bound to the extracellular matrix and get released as cartilage is degraded [33]; the release of small, leucine-rich proteoglycans and other potential damage-associated molecular pattern molecules that can directly stimulate fibroblast-like cells via Toll-like receptors; and perhaps also direct secretion of chemokines, cytokines, growth factors, and by chondrocytes. Moreover, some of these effects may be direct, whereas others may be indirect in that factors released from cartilage stimulate neighboring cells in the synovial membrane, which in turn attracts fibroblasts from the circulation [34, 35]. Our in vitro studies using MCP-1 failed to demonstrate an enhancing effect of this chemokine on SF transmigration, but there are a variety of other chemokines that may well play a role in this process. Also, it needs to be emphasized that most likely it is not only one single factor that mediates the trafficking of fibroblasts into predamaged joints, but that several of the aforementioned mechanisms contribute to this process.

## Conclusions

Currently, not much is known about pathological changes that initiate and drive the progression of RA in very early stages of the disease, despite a consensus that there are molecular and cellular alterations, including changes in cartilage structure, that precede the onset of synovitis in RA [33–38]. Furthermore, why certain joints are more likely to be affected than others remains almost completely unexplored, although there are some data derived from microanatomical studies suggesting that mechanically exposed areas in joints are more prone to developing synovitis [39, 40]. In the present study, we provide evidence that structural cartilage changes that may be present in very early phases of the disease facilitate the homing of SFs and thus promote the spread and progression of RA. On the basis of these data, we suggest that changes in cartilage homeostasis are not only a result but also a potential trigger of joint destruction in RA. As a result, early treatment of cartilage degradation may help prevent the progression of RA and its spread to previously unaffected joints. Although many questions about the responsible molecules and pathways remain unanswered, we hope that our demonstration of SF homing into predamaged joints in this work will stimulate further research in this area and eventually help to solve the conundrum of disease pattern and effects on joints in RA.

## Abbreviations

b.End5: Brain endothelioma 5 cell line
Cy.7: Cyanine 7
DiR: 1,1′-dioctadecyl-3,3,3′,3′-tetramethylindotricarbocyanine iodide
FCS: Fetal calf serum
FRI: Fluorescence reflectance imaging
hTNFtg: Human tumor necrosis factor–transgenic
IA: Intra-articular
ICAM-1: Intercellular adhesion molecule 1
IL-1: Interleukin-1
IV: Intravenous
JAM-C: Junctional adhesion molecule, C variant
MCP-1: Monocyte chemoattractant protein-1
RA: Rheumatoid arthritis
RASF: Rheumatoid arthritis synovial fibroblast
SCID: Severe combined immunodeficiency
SF: Synovial fibroblast
VCAM-1: Vascular cell adhesion molecule
VLA-1: integrin α_1_β_1_
WT: Wild type
